# Correction: A simple and traceless solid phase method simplifies the assembly of large peptides and the access to challenging proteins

**DOI:** 10.1039/c7sc90046e

**Published:** 2017-07-07

**Authors:** N. Ollivier, R. Desmet, H. Drobecq, A. Blanpain, E. Boll, B. Leclercq, A. Mougel, J. Vicogne, O. Melnyk

**Affiliations:** a UMR CNRS 8161 CNRS , Université de Lille , Institut Pasteur de Lille , 1 rue du Pr Calmette , 59021 Lille Cedex , France . Email: oleg.melnyk@ibl.cnrs.fr

## Abstract

Correction for ‘A simple and traceless solid phase method simplifies the assembly of large peptides and the access to challenging proteins’ by N. Ollivier *et al.*, *Chem. Sci.*, 2017, DOI: ; 10.1039/c7sc01912b.



## 

The authors regret that entries 2 and 3 in Table 1 are incorrect in the original manuscript. Entry 2 is missing the CH_2_CH_2_O group and entry 3 is missing two carbonyl groups.

A corrected version of Table 1 has been presented below:

**Table 1:** Linker strategies for solid phase protein synthesis in the N-to-C direction

Entry	Functional linker (FL)	Attachment method for peptide segment 1	Latent thioester (LT)		Cleavage	Ref.
			Structure	Activation method		
1	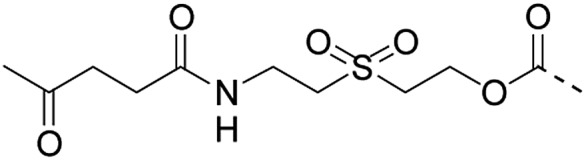	Oximation pH 4	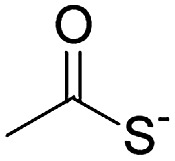	Alkylation with bromoacetic acid, pH 4.6	β-Elimination, aqueous base, pH 13	22
2	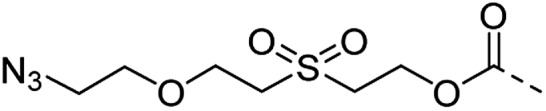	CuAAC pH 7 or SPAAC pH 2	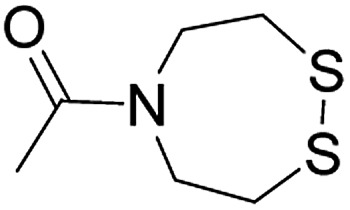	Reduction (TCEP) and exchange by 3-mercaptopropionic acid, pH 4	See entry 1	40
3	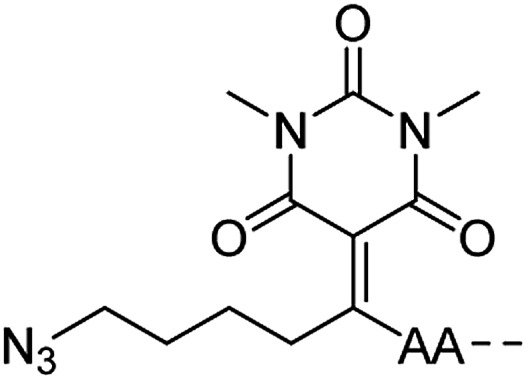	CuAAC	Note^*a*^		Transimination, 1 M H_2_NOH, pH 7–8.5	44
4 (This work)	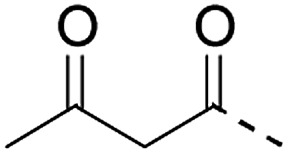	Oximation pH 3–4	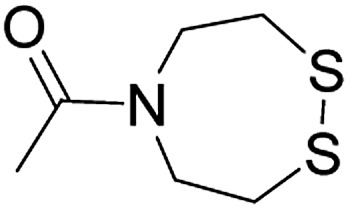	See entry 2	Transoximation 0.025 M H_2_NOH, 3 M aniline, pH 3	

^*a*^The method was used for the synthesis of large protein mimetics through CuAAC ligation.

The Royal Society of Chemistry apologises for these errors and any consequent inconvenience to authors and readers.

